# SARS-CoV-2 infectivity and antigenic evasion: spotlight on isolated Omicron sub-lineages

**DOI:** 10.3389/fmed.2024.1414331

**Published:** 2024-08-29

**Authors:** Aldo Barrera, Constanza Martínez-Valdebenito, Jenniffer Angulo, Carlos Palma, Juan Hormazábal, Cecilia Vial, Ximena Aguilera, Pablo Castillo-Torres, Catalina Pardo-Roa, María Elvira Balcells, Bruno Nervi, Nicole Le Corre, Marcela Ferrés

**Affiliations:** ^1^Departamento de Enfermedades Infecciosas e Inmunología Pediátricas, Escuela de Medicina, Pontificia Universidad Católica de Chile, Santiago, Chile; ^2^Facultad de Ciencias Biológicas, Pontificia Universidad Católica de Chile, Santiago, Chile; ^3^Laboratorio de Infectología y Virología Molecular, Facultad de Medicina y Red de Salud UC CHRISTUS, Santiago, Chile; ^4^Instituto de Ciencias e Innovación en Medicina, Facultad de Medicina, Clínica Alemana Universidad del Desarrollo, Santiago, Chile; ^5^Centro de Epidemiología y Políticas de Salud, Facultad de Medicina Clínica Alemana Universidad del Desarrollo, Santiago, Chile; ^6^Departamento de Salud del Niño y el Adolescente, Escuela de Medicina, Pontificia Universidad Católica de Chile, Santiago, Chile; ^7^Departamento de Enfermedades Infecciosas del Adulto, Escuela de Medicina, Pontificia Universidad Católica de Chile, Santiago, Chile; ^8^Departamento de Hematología y Oncología, Escuela de Medicina, Pontificia Universidad Católica de Chile, Santiago, Chile

**Keywords:** SARS-CoV-2, COVID-19, variants, Omicron, isolated viruses

## Abstract

Since the SARS-CoV-2 outbreak in 2019, a diversity of viral genomic variants has emerged and spread globally due to increased transmissibility, pathogenicity, and immune evasion. By the first trimester of 2023 in Chile, as in most countries, BQ and XBB were the predominant circulating sub-lineages of Omicron. The molecular and antigenic characteristics of these variants have been mainly determined using non-authentic spike pseudoviruses, which is often described as a limitation. Additionally, few comparative studies using isolates from recent Omicron sub-lineages have been conducted. In this study, we isolated SARS-CoV-2 variants from clinical samples, including the ancestral B.1.1, Delta, Omicron BA.1, and sub-lineages of BA.2 and BA.5. We assessed their infectivity through cell culture infections and their antibody evasion using neutralization assays. We observed variations in viral plaque size, cell morphology, and cytotoxicity upon infection in Vero E6-TMPRSS2 cells for each variant compared to the ancestral B.1.1 virus. BA.2-derived sub-variants, such as XBB.1.5, showed attenuated viral replication, while BA.5-derived variants, such as BQ.1.1, exhibited replication rates similar to the ancestral SARS-CoV-2 virus. Similar trends were observed in intestinal Caco-2 cells, except for Delta. Antibody neutralization experiments using sera from individuals infected during the first COVID-19 wave (FWI) showed a consistent but moderate reduction in neutralization against Omicron sub-lineages. Interestingly, despite being less prevalent, BQ.1.1 showed a 6.1-fold greater escape from neutralization than XBB.1.5. Neutralization patterns were similar when tested against sera from individuals vaccinated with 3xBNT162b2 (PPP) or Coronavac-Coronavac-BNT162b2 (CCP) schedules. However, CCP sera showed 2.3-fold higher neutralization against XBB.1.5 than FWI and PPP sera. This study provides new insights into the differences between BA.2 and BA.5-derived variants, leading to their eventual outcompetition. Our analysis offers important evidence regarding the balance between infectivity and antigenic escape that drives the evolution of second-generation SARS-CoV-2 variants in the population.

## 1 Introduction

The evolutionary trajectory of SARS-CoV-2 has seen a succession of genetic variants that emerge and displace previous circulating strains, potentially causing large waves of infections worldwide (1). In the early stage of the COVID-19 pandemic, the virus showed discrete aminoacidic variations, with the primary fixed substitution in the viral glycoprotein spike (S) being D614G, a host-induced adaptative mutation (2–4). Previous studies using pseudoviral particles bearing the SARS-CoV-2 S protein to approximate the role of mutations, such as L452R, N484K, and N501Y, showed that these variants were capable of circumventing neutralization by the immune response elicited from prior infection (4–6).

In January 2021, the World Health Organization (WHO) stated that different lineages of SARS-CoV-2, designated as variants of concern (VOCs), were circulating with higher transmission rates compared to the ancestral virus (B.1 in PANGO nomenclature) and were associated with more severe COVID-19 cases (4, 7). VOCs displaced the previously circulating lineages either regionally, as observed with Alpha (B.1.1.7, in Europe), Beta (B.1.351, in southern Africa), and Gamma (P.1, in South America), or worldwide, as observed with Delta (B.1.617.2) and Omicron (BA.1). The unusual number of mutations in Omicron suggests it may have originated from a long-lasting infection in an immunocompromised individual, subsequently evolving in different sub-lineages through convergent evolution (8).

To reduce mortality and curb the spread of the pandemic, vaccine formulations using antigens from the ancestral virus, whether inactivated whole viruses (e.g., Coronavac by Sinovac) or RNA expressing the S protein (e.g., BNT162b2 by Pfizer-BioNTech, mRNA-1273 by Moderna), were rapidly developed and distributed worldwide (9).

In Chile, the ancestral SARS-CoV-2 virus was first detected, isolated, and sequenced in March 2020 (10). Between 2020 and 2023, ~62,000 COVID-19 fatal cases were reported, making Chile one of the most affected American countries during the early pandemic (11). The Delta variant was the VOC associated with the highest number of deaths, circulating mainly between June and December 2021 and causing an estimated 678 deaths (12). Omicron BA.1 emerged in January 2022, causing a sharp peak in cases, displacing Delta, and showing a 2-fold reduction in the case fatality rate (12, 13). The first Omicron sub-lineage, BA.1.1, appeared a couple of months later with a similar number of cases to subsequent Omicron sub-lineages. Two principal clusters of second-generation Omicron lineages, BA.2 and BA.5, were shown to co-circulate in most countries from April 2022 to June 2023 (14, 15). Initially, BA.2 and its sub-lineages, such as BA.2.3 and BA.2.12.1, circulated predominantly between April and June 2022. BA.4.1 emerged later but was rapidly displaced by various BA.5-derived sub-lineages, including BA.5.1, BA.5.2, and BF.31 (13). The BA.5-derived BQ.1.1 linage, detected in Chile in October 2022, infected a small group of individuals (12) and was promptly outcompeted by the emergence of the recombinant BA.2-derived XBB lineage, a more transmissible but less virulent version of Omicron (16–18).

At the time of Omicron's emergence, 83% of the Chilean population was vaccinated with at least two doses of Coronavac or BNT162b2, and 57% had received a booster dose of Coronavac, BNT162b2, or ChAdOx1 (Oxford/AstraZeneca) to counteract the loss of neutralization against Omicron (13, 19). Chile has been recognized as one of the most successful nations worldwide in implementing SARS-CoV-2 vaccination programs, immunizing more than 90% of the targeted population in a relatively short period (20).

The phenotypic characteristics of Omicron differ from those of previous SARS-CoV-2 variants. The ancestral D614G viruses and VOCs such as Gamma and Delta showed an ACE2 receptor/TMPRSS2 protease-associated tropism for the lower respiratory tract, whereas original Omicron lineages bypass TMPRSS2 and target the upper respiratory tract (21). The mutational profile of Omicron was initially associated with a reduced affinity for ACE2 and lower replication rates. However, the most recent circulating sub-lineages have shown a recovery in viral fitness (22, 23). Despite this, Omicron sub-lineages have displayed sustained antibody evasion capacity, which has been shown mainly by the reduction in neutralization of multiple mutated S pseudoviruses using monoclonal antibodies and immune sera that neutralize previous variants (6, 18, 24). Authentic isolated viruses have shown a similar pattern of antibody evasion (25).

Studying whole circulating viruses is crucial for assessing infectious particle production, tropism, and antigenic properties of non-S protein mutations. While the main changes in replication capacity and antibody evasion between the original Omicron and past VOCs have been well-characterized (4), there is still a lack of comprehensive analysis of these factors among the most recent Omicron sub-lineages. The present study provides new insights into the molecular characteristics of different isolated SARS-CoV-2 variants. We show that recently circulating Omicron sub-lineages present different replicative phenotype and antibody evasion relative to the original Omicron lineages and the ancestral SARS-CoV-2 virus, especially comparing the BA.5 lineage variant BQ.1.1 and the BA.2-derived clade XBB.

## 2 Materials and methods

### 2.1 Cell culture

African Green monkey kidney cells Vero E6 (CRL-1586, ATCC), Vero E6 cells expressing Transmembrane Protease, Serine 2 (TMPRSS2) (kindly provided by Dr. Adolfo García-Sastre, Department of Microbiology, Icahn School of Medicine at Mount Sinai, New York, NY, USA.), and human colorectal adenocarcinoma cells Caco-2 (kindly provided by Dr. Marcelo López, Laboratorio de Virología Molecular, Pontificia Universidad Católica de Chile) were maintained in Dulbecco's Modified Eagle Medium (DMEM) (11965092, Gibco) supplemented with 10% heat-inactivated fetal bovine serum (FBS) (10437-028, Gibco) or 20% for Caco-2, 10 mM Sodium pyruvate (11360-070, Gibco), 1X MEM non-essential amino acids (11140-050, Gibco), 1% Pen-Strep-Neo antibiotic solution (15640-055, Gibco), and 25 μg/mL amphotericin B (15290018, Gibco). Vero E6-TMPRSS2 cells were selected with 10 μg/mL Puromycin (A11138-03, Gibco). All cell lines were grown at 37°C in a humidified incubator with 5% CO_2_.

### 2.2 Isolation and titration of SARS-CoV-2

Anonymized/encoded nasopharyngeal SARS-CoV-2 RT-qPCR-positive samples (Ct < 30) were kindly donated by the Laboratorio de Infectología y Virología Molecular, Red de Salud UC CHRISTUS, under the use approval from the Research Ethics Committee, Facultad de Medicina, Pontificia Universidad Católica de Chile (ID 210804007). A total of 100 microliters of each sample were pre-incubated 1:1 with DMEM containing 2X PSN and then inoculated into 90% confluent Vero E6 or Vero E6-TMPRSS2 (for Omicron lineages) cells in DMEM 2% FBS. The cell cultures were observed until visible cytopathic effect (CPE), and second passage stocks were obtained 5 days post-infection (dpi). RT-qPCR-positive supernatants were titrated using the plaque assay method (26), incubating serial dilutions in confluent Vero E6 cells with 1X Eagle's Minimum Essential Medium (EMEM) supplemented with 2% FBS, 021% BSA, 1% PSN, 1X L-Glutamine (A29168-01, Gibco), 10 mM HEPES (15630-080, Gibco), 0.24% NaHCO_3_, and 2% Ultrapure LMP Agarose (16520-050, Invitrogen). After 48 h, cells were fixated with 4% paraformaldehyde (PFA) overnight at 4°C and stained with 0.4% crystal violet (548-62-9, Sigma-Aldrich) for plaque-forming units (PFU) counting. All infection assays were performed in a Biosafety Level-3 laboratory.

### 2.3 Variant identification and sequencing

The SARS-CoV-2 genomic identity from each original sample and viral culture stock was determined by RT-qPCR using probes targeting variant-related mutations as described in (27), coupled with next-generation sequencing. A total of 16 samples were sequenced using the MinION Nanopore platform, employing the ARTIC SARS-CoV-2 whole-genome amplicon-based Oxford Nanopore Technologies (ONT) pipeline on a MinION^TM^ 1KC Sequencer (ONT). Briefly, samples were amplified using the ARTIC V3-V4 primer. Then, the Native barcoding protocols EXP-NBD196 and SQK-LSK109 were employed to build the amplicon libraries according to the manufacturer's instructions, as detailed in (28). The remaining samples were sequenced using Illumina WGS technology with the Nextera DNA Flex Library Prep Kit on a MiSeq sequencer, as detailed in (29).

For each sequence, the clade and lineage were identified according to the nomenclature of Nextstrain and Pangonlin, respectively (30). Finally, complete genomes with >95% coverage were uploaded to GISAID (Table 1). The nomenclature used for each isolate is based on the designation by Pango v.4.3.1 PANGO-v1.23 (31).

**Table 1:** SARS-CoV-2 isolated variant identification.

**SARS-CoV-2 variant (like)**	**Spike mutations identified by RT-qPCR**	**Original (P0) sequence GISAID name**	**Isolate (P2) sequence GISAID name**	% of identity (nucleotides)^*^	**Viral titer (PFU/mL)**
B.1.1 (Wuhan-like)	nd	hCoV-19/Chile/RM-CMM-A2P533884024/2020	hCoV-19/Chile/RM-PUC_MVL_3648/2020	100 (0)	1.8 × 10^8^
B.1.617.2 (Delta-like)	L452R, T478K	hCoV-19/Chile/VA-PUC_MVL_3649/2021	hCoV-19/Chile/RM-PUC_MVL_1346/2021	99.9 (1)	2.4 × 10^7^
BA.1.1 (BA.1-like)	D69-70, K417N, T478K, N501Y, P681H	hCoV-19/Chile/RM-PUC_MVL_2141/2021	hCoV-19/Chile/RM-PUC_MVL_2433/2021	99.9 (1)	5.6 × 10^5^
BA.2.3	K417N, T478K, N501Y, P681H	hCoV-19/Chile/RM-PUC_MVL_2621/2022	hCoV-19/Chile/RM-PUC_MVL_3345/2022	100 (0)	2.0 × 10^6^
BA.2.12.1	K417N, L452Q, T478K, N501Y, P681H	nd	hCoV-19/Chile/RM-PUC_MVL_3625/2022	nd	2.8 × 10^6^
BA.4.1 (BA.4-like)	K417N, L452R, T478K, N501Y, P681H	hCoV-19/Chile/VS-PUC_MVL_2769/2022	hCoV-19/Chile/VS-PUC_MVL_3347/2022	100 (0)	4.5 × 10^5^
BF.31 (BA.5-like)	K417N, L452R, T478K, N501Y, P681H	hCoV-19/Chile/VS-PUC_MVL_2765/2022	hCoV-19/Chile/VS-PUC_MVL_3346/2022	100 (0)	1.6**x**10^6^
BF.31.1	nd	hCoV-19/Chile/RM-PUC_MVL_3647/2023	hCoV-19/Chile/RM_SVR-P2/2023	99.9 (4)	2.5 × 10^6^
BQ.1.1	nd	hCoV-19/Chile/RM-PUC_MVL_3649/2023	hCoV-19/Chile/RM_91962-P2/2023	99.9 (4)	1.9 × 10^6^
XBB.1.5.13 (XBB.1.5-like)	nd	hCoV-19/Chile/RM-PUC_MVL_3648/2023	hCoV-19/Chile/RM_91780-P2/2023	99.9 (1)	1.3 × 10^5^

^*^Percentage of identity between genomic sequence from the original sample (P0) and a second passage isolate (P2).

nd, non-determined.

### 2.4 Infectivity, immunofluorescence, and cytotoxicity assays

For replicative kinetics, Vero E6-TMPRSS2 cells were infected with each isolate variant at MOI of 0.0001 and incubated in DMEM with 2% PBS. Aliquots of supernatants of the infection were collected at 1, 8, 24, 30, 46, 54, and 72 h post-infection (hpi) for titration. Additionally, 200 μL of the supernatant was mixed with 200 μL of lysis buffer for RNA extraction. For infections in the presence of neutralizing sera, an MOI of 0.001 of each isolate variant was preincubated for 1 h at 37°C with either pre-pandemic control sera or sera with a high SARS-CoV-2 neutralization titer (32), at dilutions of 1:3,840 or 1:1,920. The mixture was then incubated with Vero E6-TMPRSS2 cells in DMEM with 2% FBS, and supernatants were collected at 1, 12, and 24 hpi. The infective particles were titrated using the Tissue Culture Dose 50 (TCID50) method (33).

For the immunofluorescence assays, Vero E6-TMPRSS2 cells were infected and cultured on coverslips for 48 h, then fixed, permeabilized, and stained with a mouse IgG anti-SARS-CoV-2-nucleoprotein (NP) primary antibody (MA529981, ThermoFisher) diluted 1:250, followed by a goat anti-mouse IgG Alexa Fluor 488 secondary antibody (A11001, ThermoFisher) diluted 1:5,000, and DAPI (D1306, ThermoFisher) diluted 1:1,000.

Cytotoxicity was quantified using the Neutral Red Assay Kit (ab234039, Abcam) according to the manufacturer's protocol. Briefly, 24-well plates with Vero E6-TMPRSS2 cells were infected with an MOI of 0.001 for each variant. Viable cells were stained with Neutral Red 1X at 48 hpi, and the solubilized dye was measured at 492 nm.

### 2.5 RNA detection by RT-qPCR

Total RNA was extracted using the Total RNA Purification Kit in 96-well plate format (24380, NORGEN BIOTEK) according to the manufacturer's protocol. For SARS-CoV-2 RNA detection by RT-qPCR, 5 μL of extracted RNA was mixed with the LightMix SARS-CoV-2 RdRP plus EAV control kit (40-0777-10, TIB MIOLBIOL) according to the manufacturer's protocol and run on a LightCycler 480II (Roche). For viral RNA quantification, a SARS-CoV-2 Standard (COV09, Exact Diagnostics) was extracted, and a serial dilution of 1:10 (providing a linear range of quantification between 1.6 × 10^3^ and 2 × 10^5^ copies/ml) was run in parallel with the samples.

### 2.6 Neutralization assays

Three groups of 20 sera samples each were challenged against each viral isolate. The first group of samples was obtained from SARS-CoV-2 first-wave infected (FWI) individuals, collected between May and September 2020 for a study on convalescent plasma treatment (32). The second and third groups of sera, also from a previous study (34), were obtained from individuals vaccinated with two doses and one booster of BNT162b2 (Pfizer-BioNTech) mRNA vaccine (PPP) or two doses of inactivated SARS-CoV-2 vaccine (Coronavac) followed by a BNT162b2 booster (CCP). The FWI and CCP/PPP groups of samples were selected based on high S-pseudovirus neutralizing antibody titers as previously described in (32) and (34), corresponding to 1-month post-symptom onset for FWI (29.5 days average) or 1 month after the last vaccine dose for CCP/PPP (27.9 and 27.3, respectively). Sera were inactivated for 1 h at 56°C, and dilutions ranging from 1:10 to 1:81,920 were preincubated with 140 PFU of each variant, then incubated in Vero E6-TMPRSS2 cells in DMEM with 2% FBS. Plates were fixated at 5 dpi with 4% paraformaldehyde at 4°C overnight and stained with 0.4% Crystal violet to determine the inhibitory dose 50 (ID50) (35). The results are expressed as the geometric mean titer (GMT) of ID50 and the fold change of neutralization.

### 2.7 Software and statistical analysis

A phylogenetic tree was calculated using the Maximum likelihood method (UFboostrap 1000) in IQ-TREE (v2.2.2.6) (36) and FigTree (v1.4.4) software, using the sequenced SARS-CoV-2 genomes from the original samples (P0) and isolates (P2) for each variant analyzed, along with 292 GISAID sequences reported from Chile representing each variant clade circulating until March 2023. For mutation identification and prevalence, we used the GISAID EpiFlu^TM^ and outbreak.info databases (30). Immunofluorescence images were analyzed using ImageJ 1.53t software. Statistical analysis was performed using Graphpad Prism version 6 (Graphpad Software, La Jolla, California, USA). Direct comparisons were made using a two-tailed *t*-test, while multiple group comparisons were performed using a two-tailed one-way or two-way ANOVA *t*-test with a 95% CI. Statistical significance was set at a *p*-value of < 0.05.

## 3 Results

### 3.1 Identification of SARS-CoV-2 isolated variants

**Figure 1:**
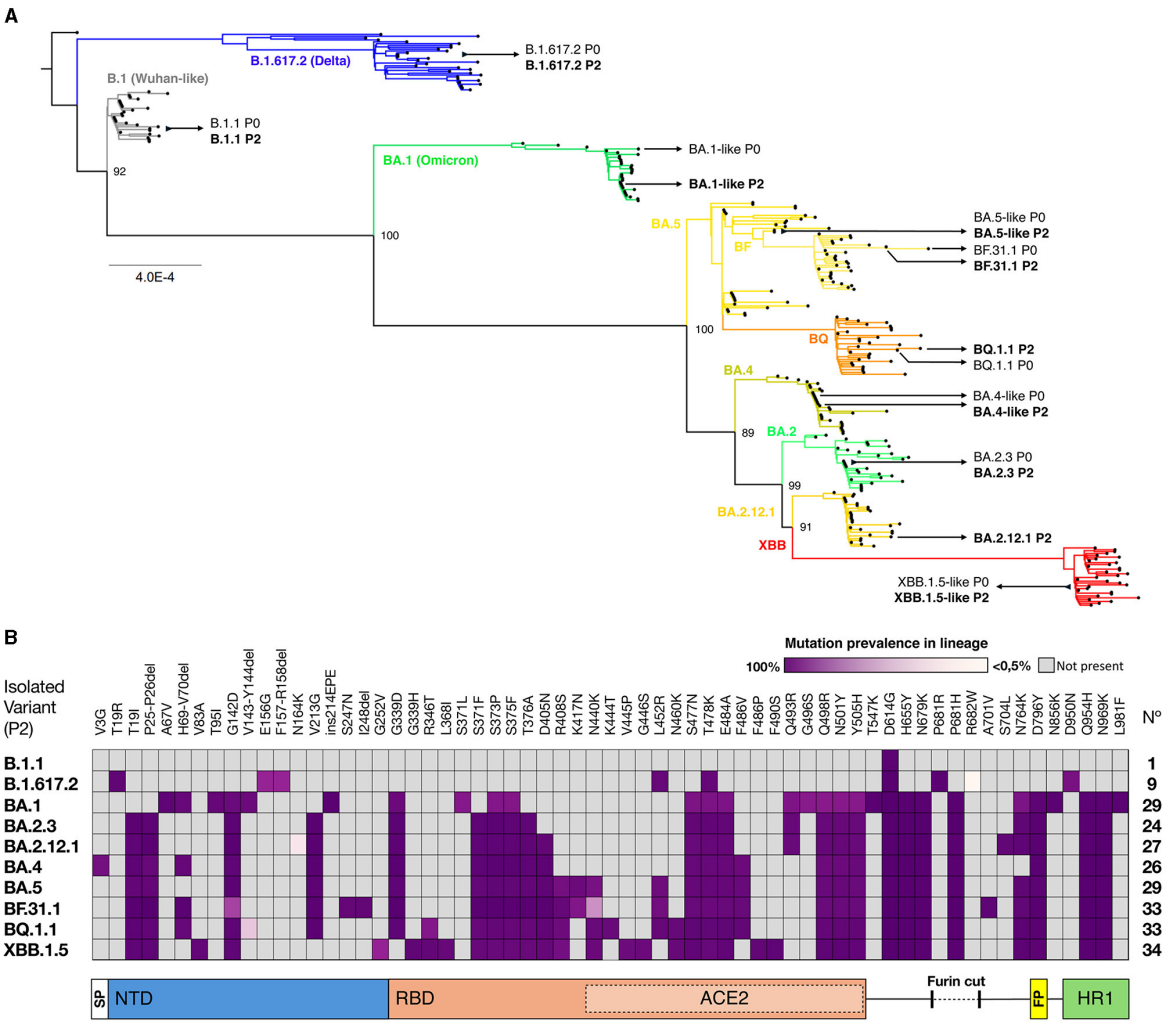
Genomic identification of SARS-CoV-2 isolates. **(A)** A maximum likelihood phylogenetic tree was performed using the SARS-CoV-2 variant genomes from the selected original samples (P0) and isolates (P2), aligned with 292 sequences from representative SARS-CoV-2 lineages circulating in Chile until March 2023. The GISAID virus names are indicated next to each assigned clade. The scale bar shows genetic distance (substitution per nt position). **(B)** Spike mutations in SARS-CoV-2 isolates. The amino acid substitutions and their prevalence in each clade were identified using GISAID databases and confirmed by alignment. Each column represents a mutation, and the spike protein domains. SP, Signal Peptide; NTD, N-Terminal Domain; RBD, Receptor Binding Domain; ACE2, region of direct contact with the ACE2 receptor; FP, Fusion Peptide; and HR1, Heptad Repeat 1 are depicted below the panel.

From a collection of SARS-CoV-2 isolates obtained between August 2020 and January 2023, 10 isolates were selected based on (i) representation from different variant clades through lineage identification and (ii) genomic identity between the original sample and the working stock passage (Table 1). We performed a phylogenetic analysis using both original and isolated genome sequences, along with genomes from GISAID representative of each cluster of circulating variants in Chile, to determine their variant identity, as shown in Figure 1A. In chronological order of isolation, a Wuhan-like B.1.1 strain bearing only the D614G S mutation was used as the ancestral SARS-CoV-2 virus to compare with isolates Delta B.1.617.2 (Delta-like), Omicron BA.1.1 (BA.1-like), BA.2.3, BA.2.12.1, BA.4.1 (BA.4-like), BF.31 (BF.5-like, closest to BA.5), BF.31.1 (isolated from an immunocompromised patient), BQ.1.1, and XBB.1.5.13 (XBB.1.5-like). According to SARS-CoV-2 genomic reports (30), most of the identified S mutations in these isolates (Figure 1B) showed a prevalence in the assigned lineage of above 60%, except for four mutations: the substitution R682W in Delta (< 0.5%), N164K in BA.2.12.1 (7%), N440K in BF.31.1 (38.7%), and the deletions V143-Y144 in BQ.1.1 (18.5%).

**Figure 2:**
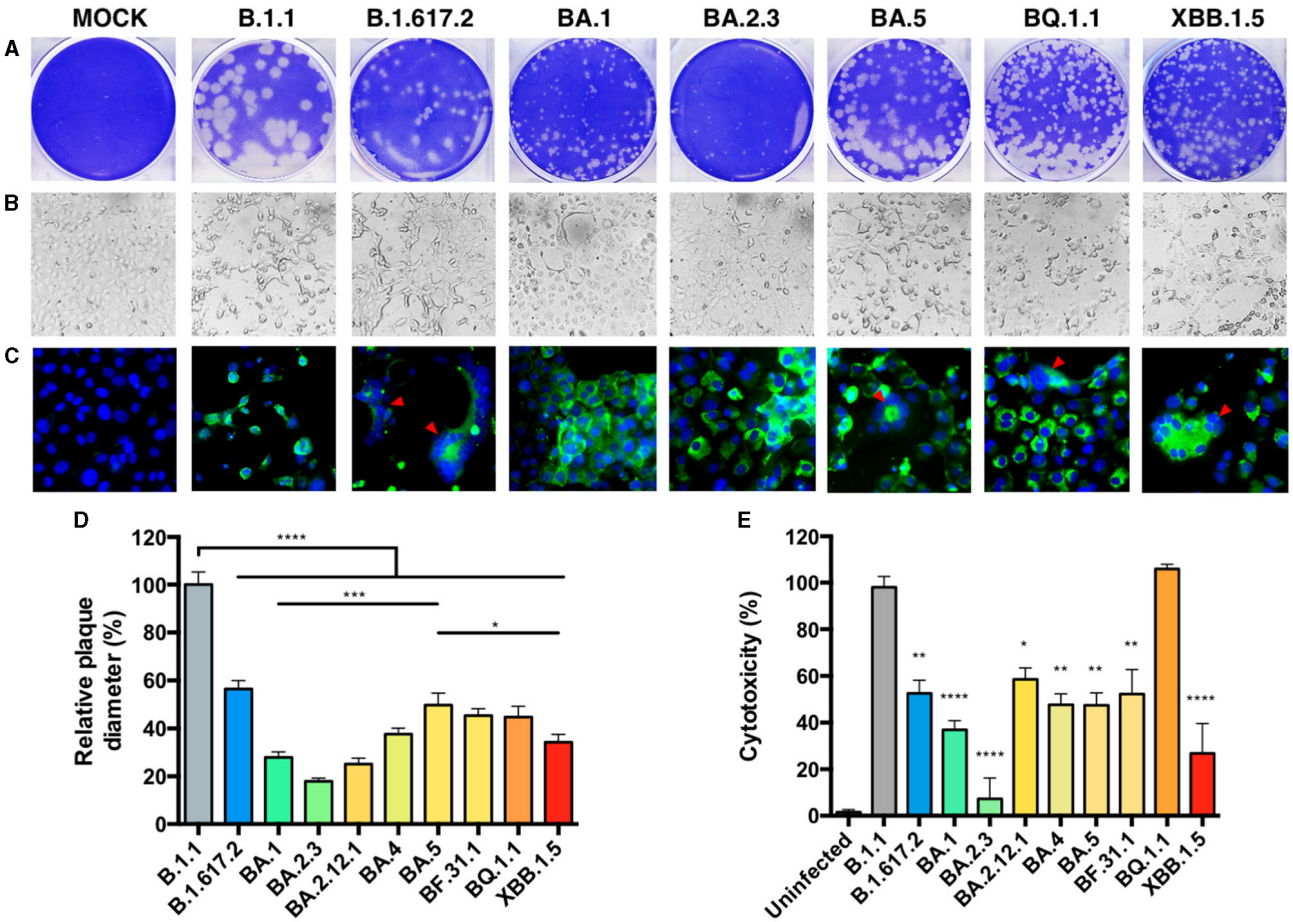
*In vitro* cytopathic effects of SARS-CoV-2 isolates. The differences in SARS-CoV-2 isolate cytopathology in a Vero E56-TMPRSS2 cell culture were observed by plaque morphology, visible cytopathic effect (CPE), and immunofluorescence (**A–C**, shown for 8 of 10 isolates and MOCK = uninfected). **(A)** Plaque assay of cells infected with serial dilutions of a second passage for each isolate was incubated for 48 h and later stained with crystal violet. **(B)** A MOI = 0.001 was used for infecting cells and observing CPE, or **(C)** an anti-SARS-CoV-2-NP staining (green) by immunofluorescence at 48 hpi. DAPI (blue) was used to observe the nucleus, and the red arrows indicated points of fusogenicity. White scale bars are equivalent to 30 μm. **(D)** The plaque diameter from 20 representative plaques in **(A)** was measured, and the percentages relative to B.1.1 were set at 100%. **(E)** The CPE was quantified by neutral red dye incorporation, and the cytotoxicity of the infection relative to an uninfected condition was set at 100%. Each bar expresses GMT and 95% CI. Statistical comparisons were performed by an ordinary one-way ANOVA for multiple comparison tests. *p < 0.05, **p < 0.005, ***p < 0.0005, and ****p < 0.00005.

We performed infection assays in Vero E6-TMPRSS2 cells, which are permissive for genomic-stable Omicron replication (37), and observed differences in plaque size (Figure 2A). The relative plaque diameter measured for the B.1.1 isolate showed the largest plaque size, followed by the Delta-like variant, while the BA.2.3 variant presented the smallest plaque, similar to the original BA.1-like, BA.2.12.1, and BA.4-like Omicron variants (Figure 2D). The three BA.5-derived variants (BF.31, BF.31.1, and BQ.1.1) showed an increase in plaque size compared to BA.1-like (*p* = 0.0003), also larger than the XBB.1.5-like plaque size (*p* = 0.0213). We also observed different monolayer disruptions for each variant at an equal MOI of input (Figure 2B). A cytotoxicity assay was performed to quantify the monolayer damage. All variants presented a reduction in cytotoxicity compared to B.1.1 infection (set to 100%), except for BQ.1.1 infection (Figure 2E), which showed higher cytotoxicity than XBB.1.5-like (*p* < 0.0005). Notably, a Spearman correlation showed no linear correlation between plaque size and cytotoxicity (Supplementary Figure S1A). To explore the distribution of infection products, we performed an anti-SARS-CoV-2-NP immunofluorescence under the same infection conditions, observing that BQ.1.1 and XBB.1.5-like infections induced a pattern of cell fusion relatively similar to Delta-like and BA.5-like, while the rest of the variants showed similar NP distribution (Figure 2C).

### 3.2 Replicative kinetics in cell culture

**Figure 3:**
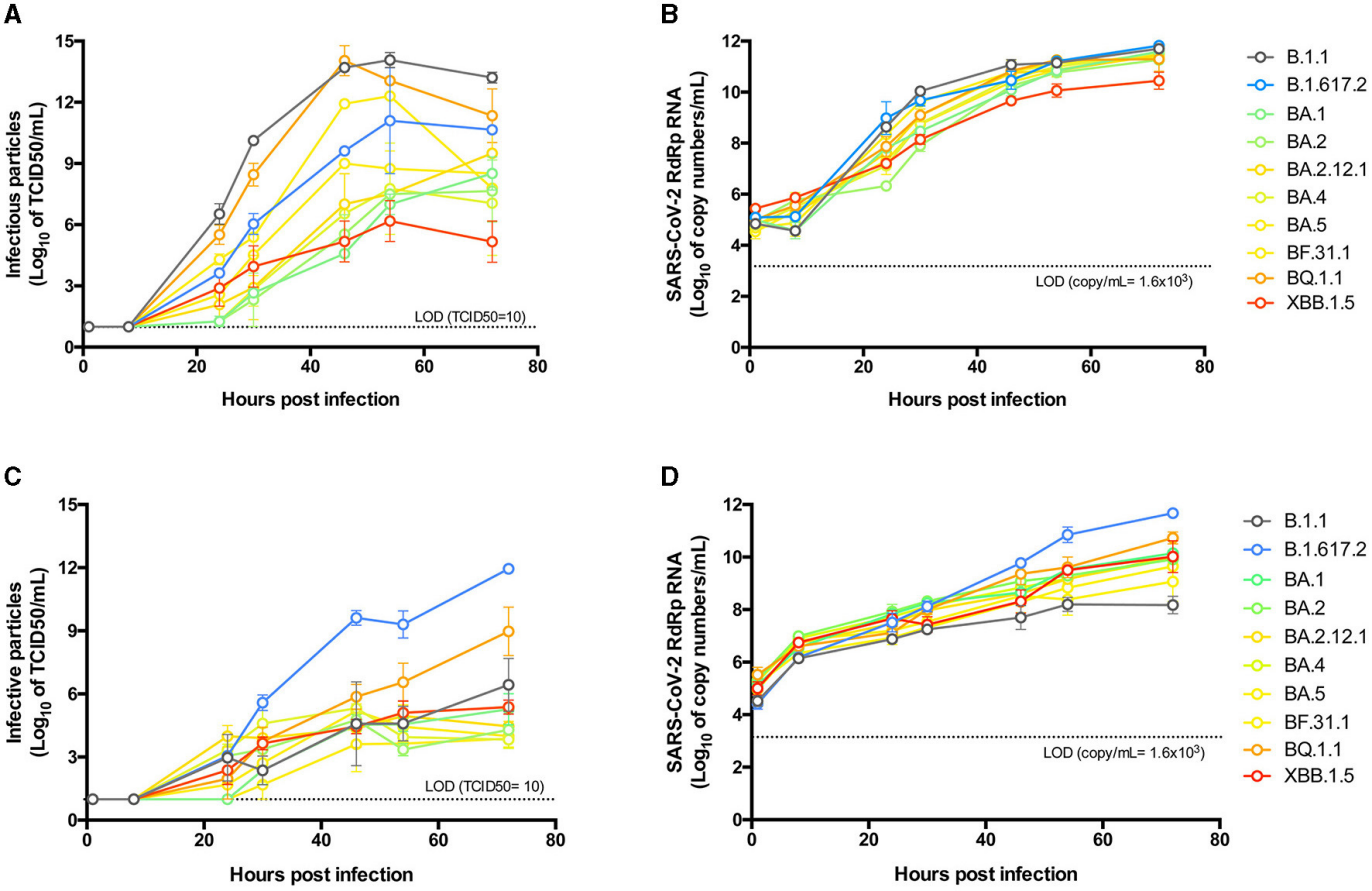
Replicative kinetics of SARS-CoV-2 isolates in cell lines. Vero E6-TMPRSS2 **(A, B)**, or Caco-2 **(C, D)** cells were infected with an MOI of 0.0001 of each SARS-CoV-2 isolate and incubated for 72 h, taking supernatant samples at 1, 8, 24, 30, 46, 54, and 72 hpi for titration of infective particles by the Tissue Culture Infectious Dose 50 (TCID50) method **(A, C)** and quantification of the RNA copy number by an RT-qPCR SARS-CoV-2-RdRp amplification assay **(B, D)**. Data points from independent experiments performed in duplicate are expressed in logarithmic scale of the mean and standard error of the mean (SEM). The statistical differences detailed in the results were determined by ordinary two-way ANOVA with Tukey's multiple comparisons test.

To study and compare the growth kinetics of the isolated variants, Vero E6-TMPRSS2 cells were infected at the same MOI, and supernatants from different times post-infection were titrated for infectious particles and viral RNA. The B.1.1 variant was shown to reach higher titers at most times post-infection, peaking at 54 hpi (Figure 3A).

Early differences were observed after 24 hpi, with the B.1.1 variant showing higher titers (*p* < 0.005) compared to BA.1-like, BA.2.3, BA.2.12.1, and BA.4-like, but not compared to Delta-like, BA.5-like, BF.31.1, BQ.1.1, or XBB.1.5-like. However, after 30 hpi, B.1.1 exhibited higher titers than all other variants except for BQ.1.1, which showed similar kinetics. Notably, BQ.1.1 showed higher infectious titers after 30 hpi compared to all BA.2-derived Omicron variants. BF.31.1 had lower titers than B.1.1 only at 30 hpi (*p* < 0.005), indicating similar kinetics to those of B.1.1 and BQ.1.1.

Interestingly, the XBB.1.5-like isolate maintained low growth kinetics, comparable to BA.1-like and BA.2-derived isolates, with a decline observed after 54 hpi. When comparing infectious titers at 46 hpi with plaque size for each isolate, we found no correlation between the two parameters (Supplementary Figure S1B). In contrast, we found a significant correlation when comparing infective particles against cytotoxicity titers (*p* = 0.0037, Supplementary Figure S1C).

To correlate the infectious particles with viral RNA, we quantified the copy number of genomic RNA produced during infection and observed more similar kinetics across all variants (Figure 3B). Notably, a correlation was found between RNA levels and infectious particles at 46 hpi (*p* = 0.0105, Supplementary Figure S1D) for all variants. RNA titers revealed differences earlier post-infection compared to infectious titers, and by 30 hpi, RNA levels for the XBB.1.5-like variant were statistically lower than those of other isolates, particularly when compared to BQ.1.1 (*p* < 0.05).

To evaluate the infectivity of each isolate in human cells, we conducted growth kinetics experiments using a colorectal adenocarcinoma Caco-2 cell line, as previous reports have demonstrated differences in the tropism of SARS-CoV-2 variants for gastrointestinal tissues (38, 39). We observed some differences compared to the results obtained in Vero cells (Figure 3C). The B.1.1 variant exhibited growth comparable to early Omicron variants but significantly lower than Delta, which showed a 5-log increase in infective particle production at 46 hpi (*p* < 0.0005). BQ.1.1 and XBB.1.5-like replicated similarly to B.1.1 at early time points post-infection but produced more viral particles at later stages, with BQ. 1.1 reaching significantly higher levels than XBB.1.5-like at 72 hpi (*p* < 0.005). The RNA production kinetics for each isolate showed a similar distribution and correlation (*p* = 0.0184) with infectious particles (Figure 3D; Supplementary Figure S1E). Compared to B.1.1, Delta presented a higher RNA copy number after 46 hpi (*p* < 0.0005). Additionally, B.1.1 reached lower RNA titers than most other isolates at 72 hpi, even lower than XBB.1.5-like (*p* < 0.0005). Unlike the previous observations, the RNA production kinetics of BQ.1.1 and XBB.1.5-like were not statistically different at any time post-infection.

### 3.3 Neutralization of isolated variants by sera from first-wave infected or vaccinated individuals

**Figure 4:**
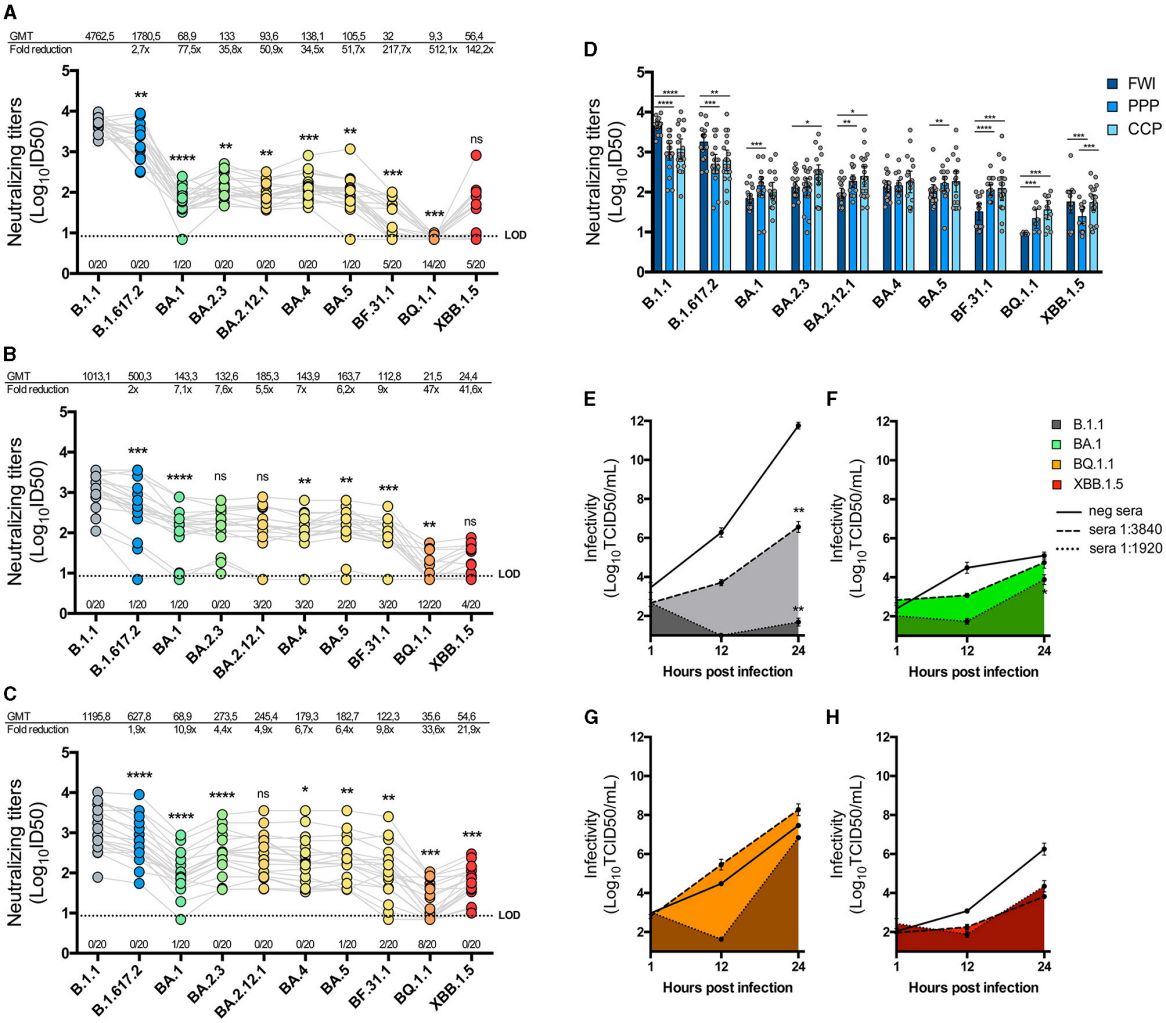
Neutralization of SARS-CoV-2 isolates by immune sera. One hundred and forty PFU of each SARS-CoV-2 isolate were pre-incubated with serial dilutions of three groups of twenty immune sera: first wave-infected individuals (FWI; **A**), individuals vaccinated with a schedule of Coronavac-Coronavac-BNT162b2/Pfizer (CCP; **B**), or individuals vaccinated with a triple BNT162b2/Pfizer (PPP; **C**) schedule. The mixture was incubated in Vero E6-TMPRSS2 cells, and 5 days later, the cells were fixated and stained to determine neutralization titers using the inhibitory dose 50 (ID50) method. The graphics show the neutralization titers on a logarithmic scale for each serum against every isolate joined by gray lines, with the number of sera that showed no reactivity against each isolate below the LOD. The geometric mean titers (GMT) and the GMT fold reduction in neutralization relative to B.1.1 are also shown in the above graphics. The successive difference between isolates' neutralization was calculated by a paired non-parametric *t*-test with a Wilcoxon's matched-paired test. Multiple comparisons are also expressed per sera group in **(D)**, with bars representing the GMT and 95% CI, and the statistical differences were determined by an unpaired non-parametric t-test with the Mann–Whitney test. **(E–H)** Replicative kinetics of B.1.1 **(E)**, BA.1-like **(F)**, BQ.1.1 **(G)**, and XBB.1.5-like **(H)** in the presence of a pre-pandemic anti-SARS-CoV-2 IgG negative sera (straight line) or neutralizing sera at dilutions of 1:3,840 or 1:1,920 (pointed lines) were performed in Vero E6-TMPRSS2 cells. The TCID50 titers were measured from supernatants collected at 1, 12, and 24 hpi and graphed in a logarithmic scale of the mean and SEM. A statistical comparison was performed using a two-way ANOVA with Tukey's multiple comparisons test. *p < 0.05, **p < 0.005, ***p < 0.0005, and ****p < 0.00005 for all tests.

We evaluated the level of immune evasion exhibited by each isolated variant against neutralizing antibodies. First, we analyzed a group of 20 sera samples from individuals infected during the first wave of SARS-CoV-2 (FWI). The results indicated that, over time, the geometric mean titer (GMT) of neutralization evasion observed for most variants was significantly different from the preceding one (Figure 4A). Compared with the B.1.1 variant, the Delta-like variant exhibited a 2.7-fold reduction in neutralization (*p* = 0.0012), while the Omicron BA.1-like variant showed a 77.5-fold reduction, which was also significantly lower than that of the Delta-like variant (*p* < 0.0001). Interestingly, the BA.2.3 variant was more susceptible to neutralization than the BA.1-like variant (*p* = 0.0017), and the BA.4-like variant was similarly more susceptible when compared with the BA.2.12.1 variant (*p* = 0.0005). The BF.31.1 and BQ.1.1 variants showed the greatest decrease in neutralization titers compared with the B.1.1 variant (217.7 × and 512.1 × , respectively), while the XBB.1.5-like variant showed an increase in neutralization compared to the BQ.1.1 variant, although this difference was not statistically significant. Furthermore, 70% of the sera samples showed no detectable reactivity against the BQ.1.1 variant.

We tested the isolated variants against other two groups of sera from individuals with different vaccination schedules: one group received two initial doses of an mRNA vaccine followed by a booster dose of the same mRNA vaccine (PPP), and the other group received two initial doses of an inactivated vaccine followed by a booster dose of an mRNA vaccine (CCP). The results for PPP sera showed varying levels of neutralization against each variant, with an overall reduction in neutralization compared to the B.1.1 variant (Figure 4B). BA.2.3 and B.2.12.1 exhibited similar levels of neutralization, as did BQ.1.1 and XBB.1.5-like variants. Notably, 60% of the PPP sera samples showed no inhibition of infection by the BQ.1.1 variant.

The results for CCP sera were similar to those for the PPP group (Figure 4C). However, BA.2.3 showed a significant increase in neutralization activity compared to the BA.1-like variant (*p* < 0.0001), as did the XBB.1.5-like variant compared to the BQ.1.1 variant (*p* = 0.0005). When comparing the neutralization effects of each group of sera in parallel, B.1.1 and Delta-like viruses were found to be more evasive to vaccination-derived sera, while Omicron sub-lineages were more evasive to FWI sera (Figure 4D). Nevertheless, XBB.1.5-like was equally neutralized by FWI and CCP sera groups but not by PPP.

To assess the effect of neutralizing sera on the replicative kinetics of isolated variants, we pre-incubated equivalent MOIs of B.1.1, BA.1-like, BQ.1.1, and XBB.1.5-like viruses (Figures 4E–H) with sera from the FWI group, which had detectable neutralization titers against each variant (B1.1 ID50 = 5,252, BA.1-like ID50 = 62.1, BQ.1.1 ID50 = 8.95, and XBB.1.5-like ID50 = 112.6). The sera dilutions used were calculated to be sub-neutralizing (1:3,840) or neutralizing (1:1,920) against B.1.1. The results showed that, compared to the infectious titers in the presence of pre-pandemic non-neutralizing control sera, B.1.1 titers decreased by 5 logs in the presence of a 1:3,840 dilution of neutralizing sera at 24 hpi (*p* < 0.005) and nearly reached the limit of detection (LOD) in the presence of a 1:1,920 dilution of sera (*p* < 0.005) (Figure 4E). However, the infectious titers of BA.1-like, BQ.1.1, and XBB.1.5-like variants in the presence of both dilutions of neutralizing sera, while showing a tendency to decrease, were not significantly affected at 24 hpi (Figures 4F–H).

## 4 Discussion

### 4.1 SARS-CoV-2 Omicron BA.5-like variants are more infective than BA.2-like variants

In this study, we analyzed the molecular properties of clinically authentic SARS-CoV-2 isolated variants, revealing significant differences in infectivity. The results of CPE and replication observed in Vero E6-TMPRSS2 cells align with previous reports, which describe more severe tissue damage in infections caused by Wuhan-like and Delta variants than those caused by Omicron. As mentioned, mutations acquired by Delta in the furin cleavage site facilitate S protein cleavage by TMPRSS2, enhancing viral fusogenicity, which is closely associated with increased viral pathogenicity (40). Delta-like variants demonstrated more successful growth than B.1.1 in Caco-2 cells, an observation that aligns with previous studies suggesting that Delta has a greater ability to infect gastrointestinal (GI) cells compared to B.1 and Omicron (39, 41). This could be explained by a higher expression of ACE2 in GI tissue (38) and the increased receptor affinity of Delta. Interestingly, BQ.1.1 also showed high replication in Caco-2, which could be related to its higher receptor affinity compared to previous Omicron variants (42). However, its relationship with GI infections has not been previously described.

In both cell lines, the Omicron variants BA.1-like and BA.2.3 exhibited reduced infectivity, which could be due to mutations present in these isolates that attenuate virulence. For example, the S mutations N856K in BA.1-like and N679K/P681H in all the isolated Omicrons variants alter the furin cleavage site, promoting endocytic entry and subsequent proteolysis by Cathepsin-L (21, 43). BA.1 receptor binding domain (RBD) mutations such as G496S and Q493R have been shown to decrease ACE-2 affinity, but these effects were reversed in BA.2 and BA.4, respectively (44, 45). The acquisition of the T376A mutation by BA.2.3 has been reported to impair viral replication by decreasing S protein processing, reducing ACE2 affinity, and its incorporation into the viral particle (46). This mutation was subsequently inherited by following Omicron sub-lineages, along with compensatory mutations.

We observed an increase in fusogenicity and infectivity in BA.5-derived sub-lineages, which is in line with previous studies showing that BA.5 causes greater disruption to the respiratory epithelium compared to the original Omicron (14, 47). Although BA.4 and BA.5 were initially identified as having identical S proteins, they differ in other viral proteins, which has been reported to impair molecular detection using RT-qPCR methods (48) the BA.5-like (BF.31) isolate differs from the BA.4-like variant by possessing a considerable number of additional S mutations, including V3G and H69-V70del in BA.4-like, and R408S, K417N, N440K, L452R, and N764K in BA.5-like.

This set of mutations, mostly related to antigenic escape, was inherited by subsequent BA.5 lineages (such as BF.31.1 and BQ.1.1) and contributed to the displacement of BA.4 (49). However, despite these significant differences, the BA.4-like and BA.5-like isolates did not show differences in CPE or infectivity.

### 4.2 SARS-CoV-2 Omicron sub-lineages present a dynamic antigenic evasion

One of the most concerning and extensively studied characteristics of emerging SARS-CoV-2 variants is their antigenic evolution. In this study, we tested the isolated variants against sera from a cohort of individuals infected with the Wuhan-like strain of SARS-CoV-2 and two cohorts who had received three doses of Wuhan antigen-based vaccine, with samples collected at similar times post-immunization.

First, the higher titers observed in the FWI cohort against the B.1.1 variant align with the well-established understanding that active infections tend to produce a stronger neutralizing antibody response compared to passive immunization (50, 51). In general, the observed decrease in neutralization against Omicron variants compared to B.1.1 and Delta-like variants is consistent with previous findings (25, 52).

Notably, BA.2.3 and BA.4-like variants exhibited reduced antibody evasion compared to their earlier circulating Omicron isolates. This phenomenon may be attributed to the reversion of the G496S mutation in BA.2, which has been observed to reduce neutralization escape and potentially enhance its effectiveness against BA.1-elicited immunity. However, this reversion has also been noted to enhance the affinity of the virus for the ACE2 receptor, a property that had diminished in BA.1 (44). Moreover, the mutation of S371L to S371F in BA.2 increases the stability of the receptor-binding domain (RBD) (53), although it may negatively impact the virus's ability to escape class 2/3 antibodies (54).

For BA.4, the acquisition of mutations D405N, F486V, and the reversion of Q493R are associated with increased infectivity and greater evasion of humoral immunity elicited by BA.1 infection but not by initial vaccination (55). Interestingly, the sera from the PPP group did not show the same effects, suggesting that the immunogenicity of the S protein might be influenced, to some extent, by other structural proteins. Several reports have shown that part of the humoral response against SARS-CoV-2 targets other structural proteins, such as N, M, and E, which may play a critical role in limiting viral infection and providing protection (56, 57).

The observed “up and down” neutralization pattern of Omicron sub-lineages by the humoral response against the original SARS-CoV-2 virus is not described when these sub-lineages are challenged with sera from Omicron-infected individuals or those immunized with Omicron bivalent vaccines (16, 58, 59). Instead, these cases exhibit a more consistent increase in antigenic evasion. This suggests that the currently circulating variants adjust their antigenicity in response to the evolving immune landscape shaped by natural infection and/or vaccination in the population.

Recent studies on variants such as BA.2.75 and BA.2.86 have shown mutation patterns that increase ACE2 affinity while reducing evasion of convalescent sera from previously circulating variants (23, 59). In fact, immunity elicited by BA.2 and BA.5 tends to target less diverse antigenic epitopes, reflecting the convergence in Omicron RBD antigenicity, which can reduce the effectiveness of vaccines against newly circulating Omicron sub-variants (6).

Moreover, more adaptive SARS-CoV-2 variants demonstrate a dynamic balance between antibody evasion and ACE2 affinity/infectivity. Variants with higher receptor affinity but lower antibody evasion—such as those in the XBB and BA.2 sub-lineages—may circulate at low prevalence until they acquire sufficient immune escape capabilities to emerge more prominently (6, 23, 58).

While most of the isolated variants in this study were identified from ambulatory patients, BF.31.1 was isolated from an immunocompromised hospitalized patient 3 months after the first SARS-CoV-2 positive test. Both BF.1 and BF.31.1 are reported to be regional variants, with 68.2 and 62% of GISAID sequences originating from Chile, respectively (30). A major drop in neutralization by the three groups of sera was observed for BF.31.1, suggesting that unique S mutations such as S247N, deletion I248, A701V, and/or NTD mutations present in this isolate—but not inherited by subsequent circulating sub-lineages—could play a critical role in immune evasion (60, 61). The study of authentic SARS-CoV-2 isolates is particularly important when assessing the characteristics of variants that evolve within a host, especially in the context of a weakened immune response. This remains one of the most widely accepted theories for the emergence of Omicron (8).

### 4.3 SARS-CoV-2 XBB lineage exhibits attenuated viral replication and evasion

We observed that BQ.1.1 exhibited higher cytopathic effects and considerably greater infectivity compared to the XBB.1.5-like variant. Fusogenicity was observed in infections caused by both BQ.1.1 and XBB.1.5-like. However, this effect was less pronounced and not associated with the lower respiratory tract tropism observed in Delta, suggesting that this effect is independent of TMPRSS2 (14, 62, 63). The emergence of mutations such as L452R (also found in Delta) and F486V increased the infectivity of BA.5-derived lineages, resulting in the displacement of previously circulating BA.2 lineages (64, 65).

XBB is a recombinant lineage between BA.2.10.1.1 and a BA.2.75-like variants (16). The F486S mutation derived from BA.2.75 is known to reduce neutralization by antibodies, but it also significantly diminishes infectivity by lowering ACE2 affinity (17, 49). Additionally, the T11A mutation in the envelope protein of XBB, which is present in the XBB.1.5-like isolate, acts as a dominant-negative substitution that reduces CPE and replication (66). Unlike BQ.1.1, XBB.1.5 does not express ORF8, which impairs its ability to inhibit the host immune response and reduce its pathogenicity (16). We also tested the infectivity of another six XBB.1.5-like isolates with different genomic backgrounds, finding that all of these variants were equally or less replicative than the compared isolate (Supplementary Figure S2).

Neutralization assays against BQ.1.1 revealed a significant drop in inhibition by FWI sera, which is consistent with reports using sera from recently immunized and/or infected groups (6, 18, 67, 68). Most of these studies show BQ.1.1 presenting comparable or even lower evasion than XBB.1.5, which differs from our observations. A previous study showed that BQ.1.1 presents less neutralization than XBB in a cohort infected with Omicron BA.1/BA.2 (69). Additionally, studies have shown that some monoclonal antibodies are more reactive against XBB.1.5 than BQ.1.1, while others show the opposite pattern (18). The F486P mutation in XBB.1.5 has been demonstrated to increase fitness compared to variants with F486S (BA.2.75) or F486V (BQ.1.1) (6, 70), but their antigenic properties are not well-described.

Despite showing reduced replication and antigenic escape from prior immunity, the outcompetition of BA.2-derived variants like XBB.1.5 over BQ.1.1 in the population is better understood by XBB.1.5′s increased transmissibility and evasion to antibodies from most recent infections (16, 71).

## 5 Conclusion

In this study, we identified unique characteristics related to the infectivity and immune evasion of isolated SARS-CoV-2 variants associated with successive waves of COVID-19 in Chile. BA.2 sub-lineages showed reduced infectivity and antibody evasion compared to BA.5 sub-lineages. However, despite these findings, only BA.2-derived variants remain prevalent in the current population, which might be attributed to a dynamic host adaptation process.

A limitation of the study is the use of immune sera that may not fully represent the current serological status of the Chilean population, where 74% of adults have received a second booster dose, and 58.8% of adults > 60 years have been vaccinated with a B.1/BA.1 bivalent boost (72). Therefore, assessing the immune escape potential of both these isolates and new circulating SARS-CoV-2 variants (such as BA.2-derived variants BA.2.86 and JN.1) against sera from representative groups is important.

Additionally, *in vivo* infection assays would better explain the implications for human health regarding the cytopathology and infectivity of the variants shown in this study. Based on this evidence, it is crucial to maintain real-time phenotypic tracking of circulating SARS-CoV-2 variants, even during periods of low-level population transmission. This approach will contribute to the coordinated global surveillance of the SARS-CoV-2 genome and help mitigate the risks of future pandemics.

## Data availability statement

The sequence data presented in the study are deposited in the GISAID repository (https://gisaid.org/), accessing with the virus names indicated in Table 1.

## Ethics statement

Ethical approval was not required for the studies on humans in accordance with the local legislation and institutional requirements because only commercially available established cell lines were used.

## Author contributions

AB: Writing – review & editing, Writing – original draft, Visualization, Validation, Supervision, Software, Resources, Project administration, Methodology, Investigation, Funding acquisition, Formal analysis, Data curation, Conceptualization. CM-V: Funding acquisition, Writing – review & editing, Validation, Supervision, Project administration, Methodology, Investigation, Conceptualization. JA: Writing – review & editing, Visualization, Supervision, Methodology, Investigation, Conceptualization. CP: Writing – review & editing, Methodology, Investigation, Formal analysis, Data curation. JH: Writing – review & editing, Resources. CV: Writing – review & editing, Resources, Funding acquisition. XA: Writing – review & editing, Resources. PC-T: Writing – review & editing, Visualization, Software, Methodology, Formal analysis, Data curation. CP-R: Writing – review & editing, Supervision, Resources, Methodology, Investigation, Formal analysis. MB: Writing – review & editing, Resources. BN: Writing – review & editing, Resources. NL: Writing – review & editing, Validation, Supervision, Resources, Funding acquisition, Conceptualization. MF: Writing – review & editing, Visualization, Validation, Supervision, Resources, Project administration, Funding acquisition, Conceptualization.

## Supplementary material

Supplementary Figure S1 **(A–C)** Correlations between parameters of SARS-CoV-2 infection. GMT data for each isolate was compared by nonparametric, two-tailed Spearman correlation with 95% CI. A line expressing the linear regression was calculated (red line for positive correlation), and Spearman *r* and *p*-values are shown for each graph. **(D–H)** Compare infectivity and RdRp RNA in Vero E6-TMPRSS2 and Caco-2 cells, respectively. ^*^*p* < 0.05. ^**^*p* < 0.005.

Supplementary Figure S2 Infectivity of different SARS-CoV-2 XBB.1.5 isolates. Vero E6-TMPRSS2 cells were infected with an MOI of 0.0001 of each SARS-CoV-2 isolate, and infectivity titers were calculated at 48 hpi. Bars indicate the GMT and 95% CI. The gray bar indicates the XBB.1.5-like isolate used in the previous assays.
