# Supplementary figures and images for: SARS-CoV-2 infectivity and antigenic evasion: spotlight on isolated Omicron sub-lineages
Source: Front Med (Lausanne). 2024 Aug 29;11:1414331. doi: 10.3389/fmed.2024.1414331 (PMC11390582; doi:10.3389/fmed.2024.1414331)

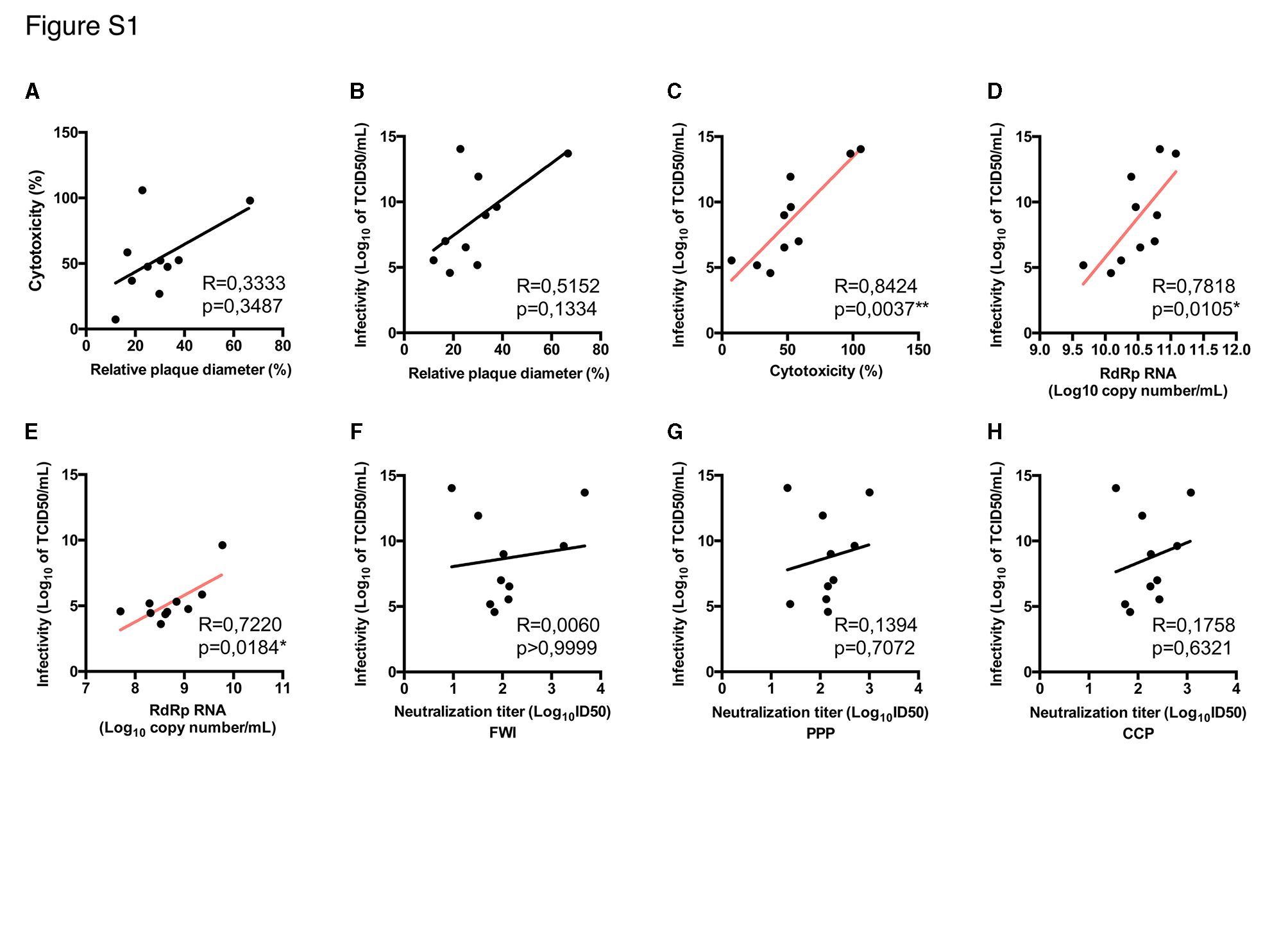

Supplement: Supplementary Figure S1 — (A–C) Correlations between parameters of SARS-CoV-2 infection. GMT data for each isolate was compared by nonparametric, two-tailed Spearman correlation with 95% CI. A line expressing the linear regression was calculated (red line for positive correlation), and Spearman r and p-values are shown for each graph. (D–H) Compare infectivity and RdRp RNA in Vero E6-TMPRSS2 and Caco-2 cells, respectively. *p < 0.05. **p < 0.005. [file Images_1.tif]

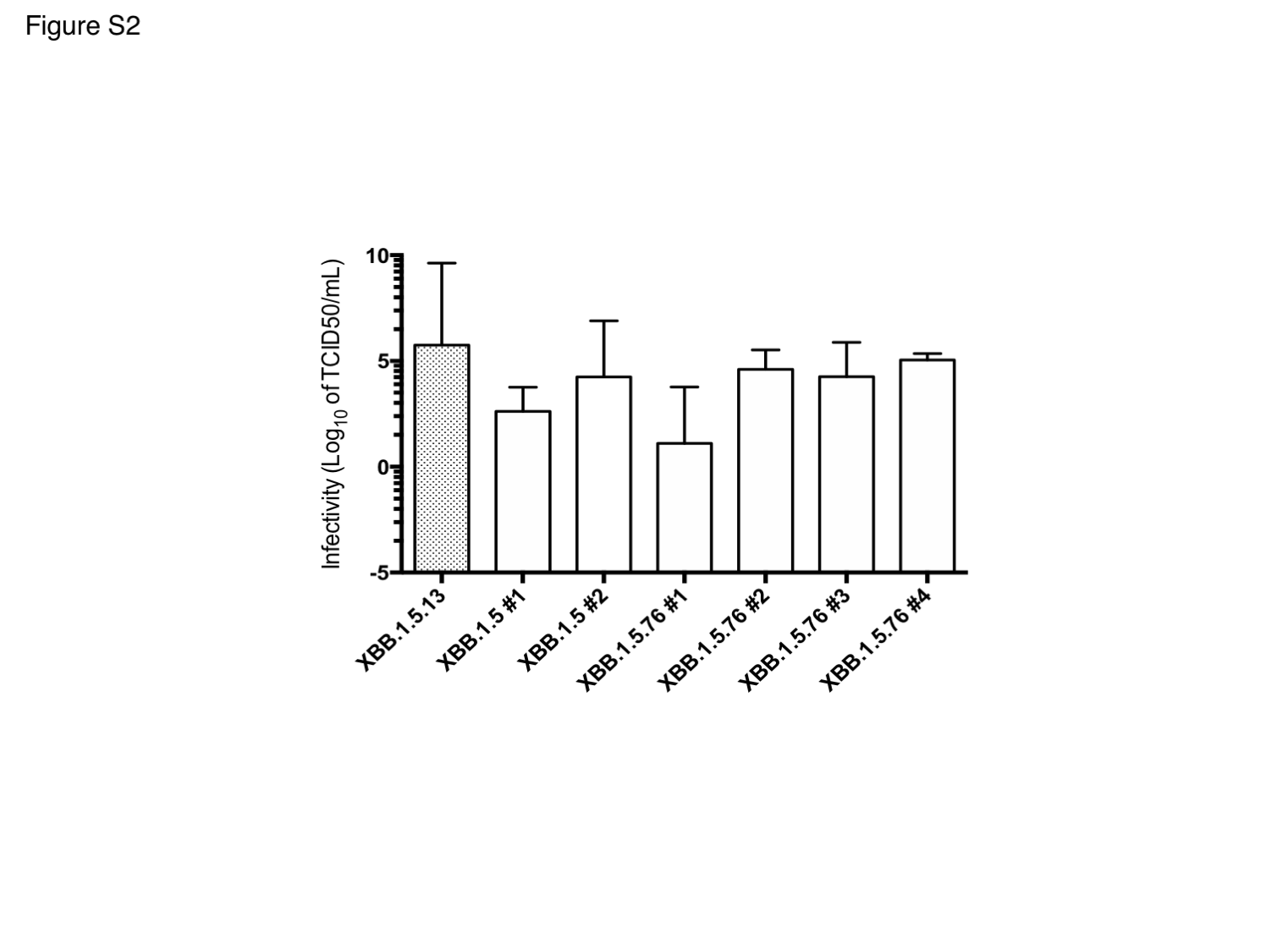

Supplement: Supplementary Figure S2 — Infectivity of different SARS-CoV-2 XBB.1.5 isolates. Vero E6-TMPRSS2 cells were infected with an MOI of 0.0001 of each SARS-CoV-2 isolate, and infectivity titers were calculated at 48 hpi. Bars indicate the GMT and 95% CI. The gray bar indicates the XBB.1.5-like isolate used in the previous assays. [file Image_2.TIFF]
